# A comparative analysis of an AI-assisted virtual reality platform with technician-based examination for visual field, visual acuity, and color vision in neuro-ophthalmology

**DOI:** 10.1007/s00417-026-07148-w

**Published:** 2026-02-16

**Authors:** Carlos E. Mendoza-Santiesteban, Amanda Tran, Henar Albertos-Arranz, Ximena Mendoza-Infante, Nicole M. Mendoza-Hung, Paul R. Clifford, Giselle Ricur, Ioannis Del-Campo, Tamara Juvier-Riesgo

**Affiliations:** 1 Bascom Palmer Eye Institute, University of Miami Health System Miami, Miami, FL USA; 2 St. George’s University School of Medicine, St. George, Grenada; 3 University of Miami, Miller School of Medicine, Miami, FL USA; 4 Florida International University, Miami, FL USA; 5 College of Medicine, University of Florida, Gainesville, FL USA

**Keywords:** Virtual reality, Head-mounted device, Visual field, Visual acuity, Color vision, Neuro-ophthalmology

## Abstract

**Purpose:**

This study aimed to compare the AI-assisted Virtual Reality Platform (VRP) with technician-assisted exams for assessing visual acuity, color vision (CV), and visual field (VF) in a neuro-ophthalmology clinic.

**Methods:**

A comparative, cross-sectional study including 59 adult patients was performed in the neuro-ophthalmology clinic at Bascom Palmer Eye Institute. The Snellen chart for visual acuity, Ishihara testing, and the SITA or AVA Fast strategy for VF testing were used in both the VRP and technician-based examinations. Best corrected visual acuity (BCVA), CV results, mean deviation (MD), pattern standard deviation (PSD), and reliability indices were collected.

**Results:**

The study included 118 eyes with a mean age of 47.2 ± 18.8 (71.19% female). Significant positive correlations were found between the VRP and technician-based examination for BCVA (*r* = .641, *P*<.001) and CV (*r* = .824, *P*<.001). Bland-Altman analysis showed good agreement between VRP and the traditional exams for BCVA (mean bias 0.09 ± 0.25, limits of agreement (LOA): [0.58] to [-0.40]) and CV (mean bias 0.07 ± 0.11. LOA: [0.29] to [-0.16]). The MD and PSD of the VRP were significantly correlated with the standard Humphrey Visual Field (HVF) exam (MD: *r* = .91, *P*<.001; PSD: *r* = .88, *P*<.001). Bland-Altman analysis revealed a mean bias of -0.71 ± 2.81 for MD and − 0.90 ± 2.08 for PSD. The VRP reduced test duration (124.31 ± 0.35 s) compared to the standard exam (256.8 ± 7.1 s, *P*<.0001).

**Conclusions:**

The VisuALL VRP, a compact portable device, effectively provides results comparable to technician-based assessments in neuro-ophthalmological patients while reducing test duration and improving clinical workflow.

## Key messages

**What is known:**

- VRP has proven valuable for assessing visual fields in glaucoma patients, as well as for ophthalmology training and evaluating patients with amblyopia.

**What is new:**

- The virtual reality platform (VRP) showed a strong correlation with standard exams for visual acuity, color vision, and visual fields in neuro-ophthalmology patients.
- The VRP significantly reduced visual field test duration, enhancing clinical efficiency and workflow.
- The VRP demonstrated high reliability and patient tolerability due to its portable and ergonomic design and the AI virtual assistant enhanced patient engagement.

## Introduction

Virtual reality (VR) devices have proven beneficial in healthcare, mainly due to their portability, minimal use of retail space, and improved ergonomics [1]. The VR systems have been implemented in ophthalmology as a training tool for surgery and for the assessment, diagnosis, and treatment of several diseases [2]. This technology enables the simulation of a real environment where immersion, sensory feedback, and interaction come together [2]. In this context, VR devices are a potential solution that circumvents the challenges and limitations of conventional vision testing modalities, allowing them to evaluate the same visual parameters as those from the technician-based clinical exams [3]. For instance, some patients, including neuro-ophthalmological patients, frequently have physical limitations and/or claustrophobia when performing VF in the standard enclosed machine [4]. Head-mounted VR devices address this limitation and improve ergonomics.

Within these devices, the VisuALL (Olleyes, Inc., Summit, NJ) is a novel, US Food and Drug Administration (FDA)-registered (Class I), artificial intelligence (AI) assisted virtual reality platform (VRP) with the capability to assess visual acuity, color vision, contrast sensitivity, pupillometry, ocular motility, and visual fields (VF) in adult and pediatric populations. An additional advantage of the VF exam on the VRP device is the ability to perform binocular testing without occluding each eye, as independent screens stimulate both eyes [3]. The AI virtual assistant within the VRP device can provide instructions in multiple languages as it monitors patients throughout the tests, optimizing the testing procedure and the clinical workflow.

Several of the previously mentioned tests are part of the classical neuro-ophthalmological exam. Visual acuity (VA) and color vision (CV) testing are relevant for the diagnosis and follow-up of neuro-ophthalmic disorders [5], given their complex etiologies and progressive nature. Furthermore, the VF exam is an invaluable part and is typically performed on stationary standard automated devices, such as the Humphrey Visual Field (HVF) [6, 7] or the Octopus perimeter [7, 8]. These devices occupy a large physical footprint and require continuous monitoring by skilled personnel. While these assessments are crucial in determining VF defects and delineating the localization of lesions along the visual pathways, many patients dislike the test as it requires prolonged positioning and induces discomfort [9]. Additionally, the machine’s limited adaptability to accommodate patients with physical limitations [10], such as those with neurological or rheumatological disorders, is a factor that precludes the examination in some patients. The time it takes to complete a VF on the HVF can range from four to ten minutes per eye [11]. The long duration of the HVF testing is attributed in part to patients’ discomfort, slowing the clinic flow and resulting in prolonged waiting times [12].

Given the constraints of traditional vision assessments, there is a pressing need for a more efficient and user-friendly alternative to technician-based examinations. Currently, no studies have evaluated any VR device analyzing different visual parameters in neuro-ophthalmological patients. Therefore, this study compares the performance of the fully automated VRP with the technician-based methods in evaluating VA, CV, and VF in a neuro-ophthalmology clinic. By doing so, our goal is to assess whether the VRP can offer comparable results while addressing the limitations associated with traditional methods.

## Methods & materials

### Study design and subjects

Cross-sectional comparative study, which includes 118 eyes from fifty-nine subjects from the neuro-ophthalmological clinic of the Bascom Palmer Eye Institute (Miami, US). The inclusion criteria were patients aged over 18 years old. Exclusion criteria included spherical refraction outside ± 5.0 D and cylinder correction outside 2.0 D, recent intraocular surgery (less than 90 days before enrollment), and the inability to use the VR device after the training session. The study was approved by The University of Miami Miller School of Medicine Institutional Review Board/Ethics Committee of the Bascom Palmer Eye Institute, which adheres to the tenets of the Declaration of Helsinki and the Health Insurance Portability and Accountability Act (HIPAA). Written informed consent was obtained from all participants before enrollment.

### Device

The VisuALL VRP (Olleyes, Inc., Summit, NJ, USA) comprises two main parts: the hardware and the software. The hardware consists of a head-mounted device (HMD), weighing 276 g (Pico Interactive, Inc., San Francisco, CA), a Bluetooth-connected controller, and a web-capable device (laptop, phone, or tablet). The HMD includes a Wide Quad High-Definition Liquid Crystal Display (LCD) with a resolution of 3840 × 2160 pixels with a refresh rate of 75 Hz and 818 pixels per inch. The display is divided into two halves or monitors (one for each eye) with a resultant resolution of 1920 × 2160 pixels on each half. The display measures 119.13 × 67.06 mm, and it is placed at a distance to subtend a field of view of up to 100 degrees. The HMD includes several eye-tracking systems, inertial measurement units consisting of gyroscopes and accelerometers, and infrared (IR) based position tracking with two arrays of 6 IR sensors. The HMD communication protocols include Wi-Fi Dual-Band (WLAN) 2.4 GHz and 5 GHz and Bluetooth 4.1. The HMD has access to replaceable face inserts that are easy to clean for mass use. The software component includes the VisuALL web application, written by the Unity algorithms (a cross-platform gaming engine; Unity Technologies, San Francisco, CA), and stored on a HIPAA-compliant cloud-based server [3].

### Testing procedures

**Fig. 1:**
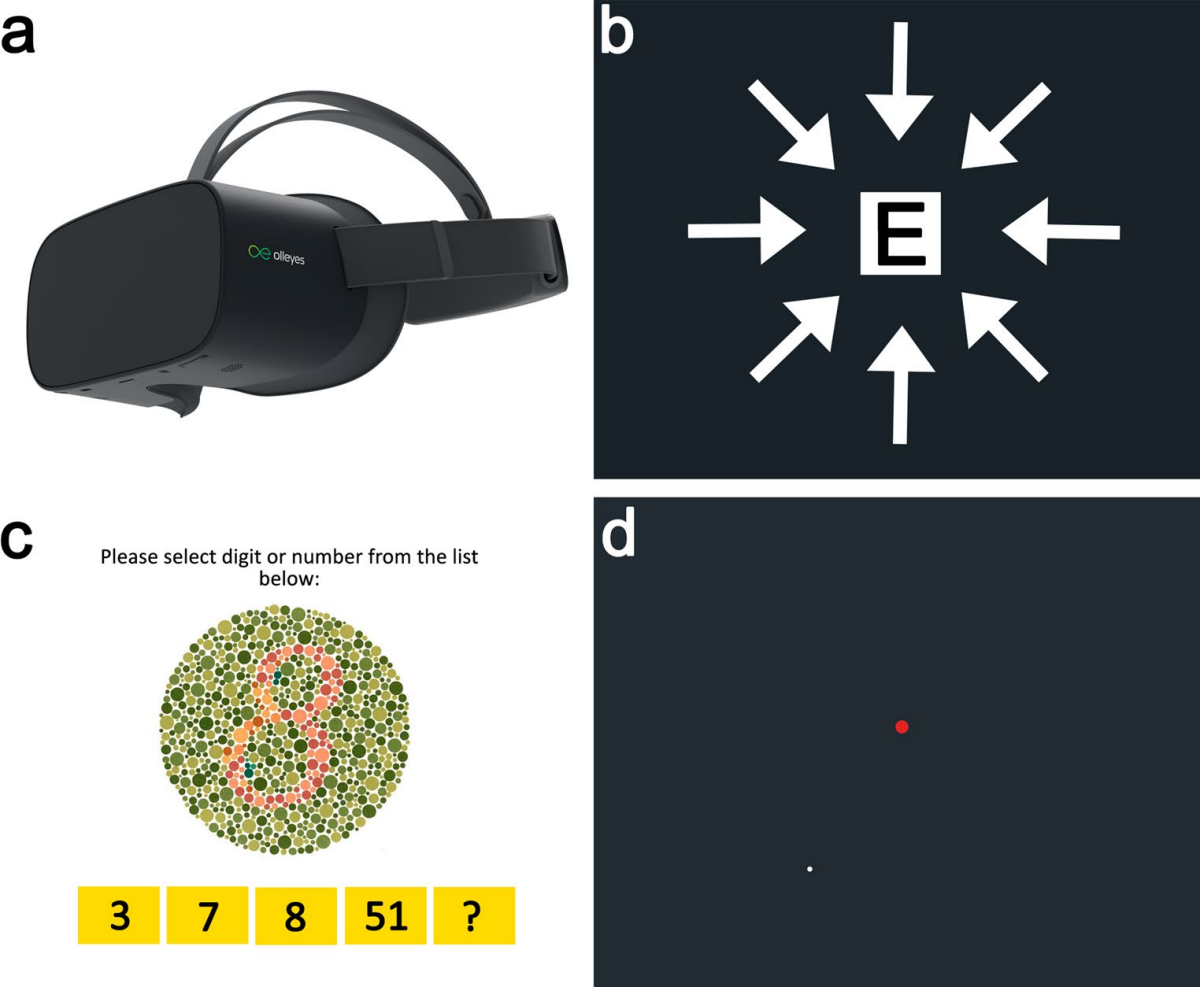
The VisuALL Virtual Reality Platform. **(a)** VR device. **(b-d)** Representative images provided by the device for (**b**) measurement of VA, (**c**) color vision testing and (**d**) VF testing. (**d**) The red dot corresponds to the fixed stimulus, while the white dot changes during the test to assess the patient’s VF

Participants underwent a technician-based ophthalmic evaluation following the neuro-ophthalmology clinic protocol. Best corrected visual acuity (BCVA) was assessed monocularly using the Snellen eye chart at 6 m. The CV was evaluated monocularly with the Ishihara booklet (11 or 14 plates, Tokyo, Japan). Visual field testing was conducted using the HVF analyzer (Zeiss Inc., Dublin, CA, USA) with the SITA Fast strategy (30 − 2 threshold, Goldmann size stimulus III, white). The same tests were repeated using the VRP device following the standard protocol. Patients wore their best correction and completed the tests while seated in the consultation room. Guidance on using the VRP device was provided to ensure optimal results, and the HMD was adjusted for each subject (Fig. 1a).

The artificial intelligence-based virtual assistant provided instructions and consistent monitoring throughout the tests. The BCVA was measured monocularly using black E Snellen letters surrounded by flanks to simulate a crowding environment, and 11 or 14 Ishihara plates were presented for each eye to CV. These tests were based on a forced-choice procedure, where participants used the controller to either indicate the aperture direction of the ‘E’ or select a number from a list in the Ishihara (Fig. 1b-c). Visual field was conducted for each eye using the AVA Fast (30 − 2 threshold, Goldmann size stimulus III, white) (Fig. 1d). This test works similarly to the standard HVF test: subjects must press the controller when they detect a spot in the visual field while maintaining focus on the central fixation target.

Visual acuity was compared using the decimal scale. Color vision results were normalized based on the total number of plates used in each test and reported as a percentage of the ratio of seen versus the total number of slides. For VF testing, the following parameters were evaluated: mean deviation (MD), pattern standard deviation (PSD), the reliability indices (including false positives (FP), false negatives (FN), and fixation losses (FL)), and test duration. Results of test reliability were assessed using previous indices and thresholds recommended in the literature: FL < 20%, FP < 15%, and FN < 33%) [13–15].

However, if two or more reliability indices also indicated an unreliable visual field in the VRP (greater than 50% for FL -, or greater than 33% for FN and for FP), the eyes were excluded from the analysis. These thresholds in the VRP are based on the previous literature [13–15]. Regarding FL, the system initially presents four stimuli within the blind spot; if the subject detects more than two of these four stimuli, fixation losses exceed 50%, and the test is therefore less reliable.

### Statistical analysis

Statistical analysis was performed using the SPSS software (IBM Statistical Package for the Social Sciences, Version 29.0.2.0). Descriptive statistics, including mean and standard deviation, were used to summarize the data. The mean difference between the technician-based exam and the VRP device was calculated for BCVA, color vision, MD, and PSD.

Bland-Altman analysis was performed to assess the agreement between the technician-based exam and the VRP for BCVA, CV, and VF MD and PSD measurements, along with Pearson’s correlation coefficient (r). The intraclass correlation coefficient (ICC) was used to assess the consistency of those parameters across the different devices. The level of agreement in the ICC was defined according to the standard criteria: excellent reliability (ICC > 0.9), good reliability (values between 0.75 and 0.9), moderate reliability (values between 0.5 and 0.75), and poor reliability (ICC < 0.5) [16]. A paired t-test was used to analyze differences in VF testing time between devices. Statistical significance was set at *P*<.05.

## Results

### Demographics

One hundred and eighteen eyes from a total of 59 participants were collected (mean age of 47.2 ± 18.8; 71.19% female). The most common ocular findings in the neuro-ophthalmology clinic were optic atrophy (22.88%), papilledema (18.64%), followed by diplopia (5.08%). In addition, 18.64% of patients presented with neurological conditions requiring ophthalmological evaluation, such as pituitary adenoma, third cranial nerve palsy, or oligodendroglioma. The remaining conditions are detailed in Table 1.

**Table 1:** Characteristics of participants

Condition or disease	Eyes, No. (%)
Optic atrophy	27 (22.88%)
* Compressive optic neuropathy*	10
* Post-NAOIN/AION*	1
* Post-Optic neuritis*	5
* Toxic-nutritional optic neuropathy*	2
* Hereditary optic neuropathy*	2
* Unknown cause*	6
* Glaucoma*	1
Papilledema	22 (18.64%)
Optic nerve edema	5 (4.24%)
* Due to optic neuritis*	3
* Due to NAION*	2
Congenital optic nerve abnormality	4 (3.39%)
Diplopia	6 (5.08%)
Other neurological conditions	22 (18.64%)
Other diseases (autoimmune, thyroid disease) w/o retinal or optic nerve involvement	10 (8.47%)
Visual disturbances/Migraine w/o retinal or optic nerve involvement	20 (16.95%)
Other retinopathies (retinitis pigmentosa)	2 (1.69%)

Optic atrophy was classified as *unknown* when the patient presented with an established optic atrophy and lacked a reliable medical history. Other neurological conditions primarily include brain tumors, blepharospasm, strokes, and facial spasms.

### Measurements of visual acuity and color vision showed good agreement between technician-based exam and VRP methods

Measurements of BCVA in decimal scale were 0.85 ± 0.28 for the technician-based exam and 0.76 ± 0.31 for the VRP device (Table 2). Nineteen eyes were excluded from the analysis due to poor patient cooperation. Specifically, up to 67.7% of patients had a difference of two lines or less when comparing the BCVA with Snellen and VR, while 32.3% had a difference greater than two lines.

**Table 2:** Results of the visual exam using technician-based exam and VRP

Parameters	Technician-based exams (*n*)	Virtual Reality Platform (*n*)	Mean differences (abs)
BCVA	0.85 ± 0.28 (99)	0.76 ± 0.31 (99)	0.09 ± 0.25
Color (correct answers)	94% (113)	88% (113)	6.73 ± 11.36
Visual field			
MD	−3.7 ± 6.15 (90)	−2.98 ± 6.89 (90)	−0.71 ± 2.81
PSD	3.97 ± 4.26 (90)	4.85 ± 4.17 (90)	−0.90 ± 2.087
FN	3.69 ± 7.96% (90)	0% (90)	-
FP	4.22 ± 5.91% (90)	0.83 ± 1.94% (90)	-
Fixation losses	14.9 ± 18.8% (89)	0.9 ± 2.1% (90)	-
Duration	256.8 ± 7.1 s	124.31 ± 0.35 s	-

Results are shown as mean ± SD. BCVA: best corrected visual acuity; MD; mean deviation; PSD: pattern standard deviation; FN: false negative; FP: false positive; abs: absolute value

**Fig. 2:**
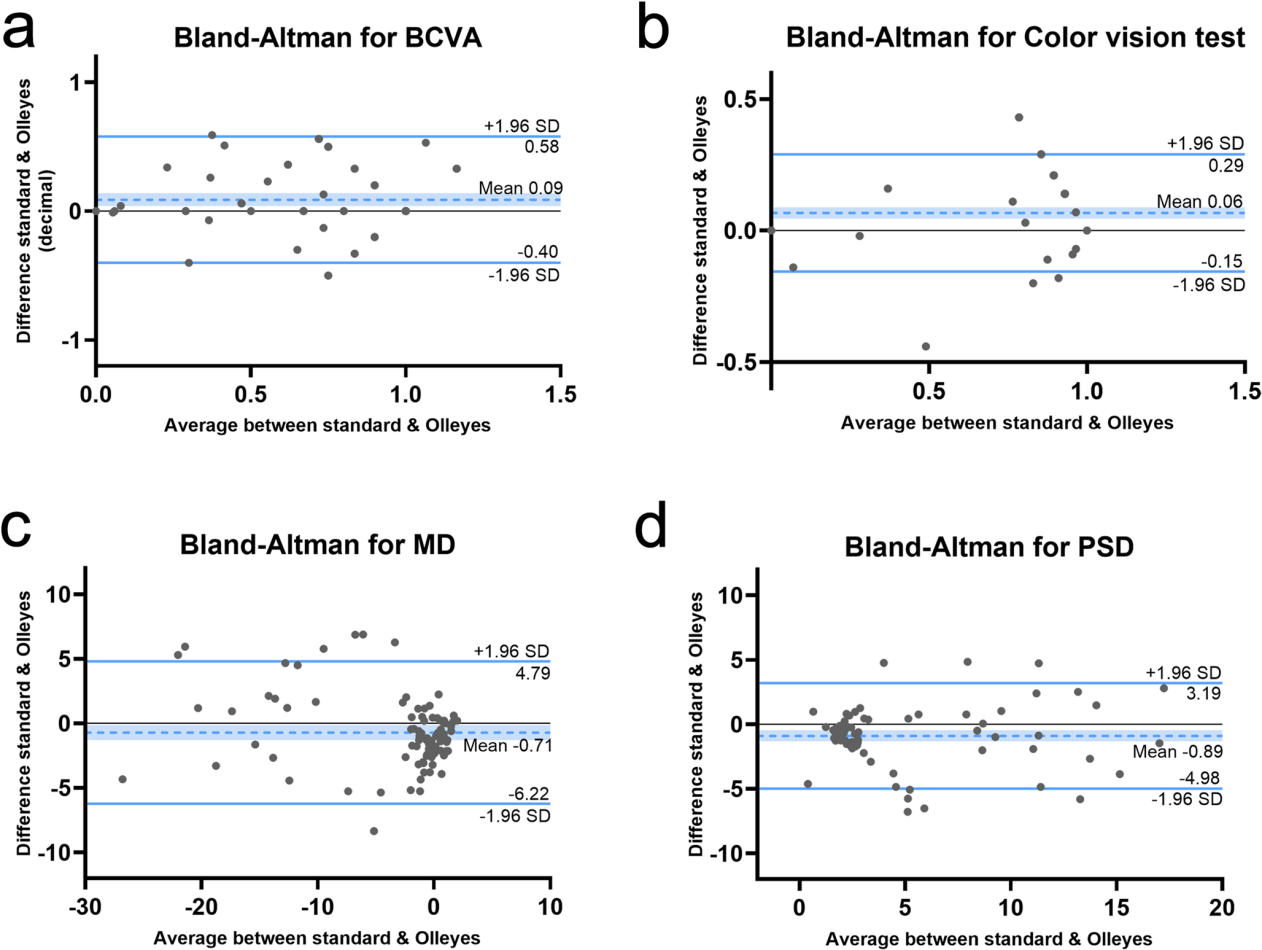
Bland-Altman plots comparing technician-based eye examinations and VisuALL VRP. (**a**) Agreement between the Snellen chart and the E Snellen test of VR device. **(b)** Bland-Altman plot showing agreement between the Ishihara test performed with the booklet and the VRP. **(c-d)** Agreement plots for MD and PSD in VF testing using the HVF and the VRP

There was a significant positive correlation of BCVA between Snellen and VRP device (*r* =.641, *P*<.001). The Bland-Altman plot showed the mean difference between the standard visual acuity exam, and the VR platform was 0.09 ± 0.25, with the limits of agreement of 0.58 and − 0.40 (Fig. 2a). Since most data points fall within this range, these results indicate a good agreement between the two methods for measuring visual acuity. Additionally, the intraclass correlation coefficient (ICC) was 0.78 (95% CI [0.67–0.85], *P*<.001), indicating good reliability between methods.

Regarding the CV, the mean percentage of correct plates was 94% for the standard Ishihara test and 88% for the same Ishihara test in the VRP. Two eyes were excluded from the analysis due to poor understanding of the test instructions, and two because one of the tests was not performed.

The color vision results obtained from the technician-based eye exam and the VRP device were significantly and positively correlated (*r* =.819, *P*<.001). Additionally, the Bland-Altman plot revealed an average difference of 0.067 ± 0.113 between the standard test and the VR platform, with the agreement limits ranging from 0.29 to −0.16 (Fig. 2b). The color measurement with the VR platform demonstrated good agreement with the technician-based eye exam, similar to the results for BCVA. Moreover, the ICC was 0.90 (95% CI [0.85–0.93], *P*<.001), indicating good reliability between both methods.

### Visual field testing and agreement between HVF and the VRP

The mean deviation (MD) of the technician-based eye examinations eye exam and the VRP was − 3.7 ± 6.15 and − 2.98 ± 6.89, respectively. The pattern standard deviation (PSD) was 3.97 ± 4.26 for the HVF exam and 4.85 ± 4.17 for the VRP (Table 2). Both measurements showed a significant positive correlation between the HVF and VRP tests. The MD had a correlation coefficient of 0.91 (*P*<.001) while the PSD had a coefficient of 0.88 (*P*<.001). Four eyes were excluded from the visual field analysis due to poor understanding of the test instructions.

The Bland-Altman plot revealed a good agreement between the HVF and the VRP tests for the MD (−0.71 ± 2.81; limits of agreement of 4.79 to −6.22) (Fig. 2c) and the PSD (mean − 0.89 ± 2.08, with limits of agreement of 3.19 and − 4.98) (Fig. 2d), as all data points (except for one) fall within the limits of agreements. The VRP appears to cause a small overestimation in the MD and the PSD in neuro-ophthalmological patients. Furthermore, the ICC for MD was 0.95 (95% CI [0.93–0.97], *P*<.001) and 0.93 for the PSD (95% CI [0.90–0.96], *P*<.001), indicating excellent reliability between methods.

Regarding the reliability indexes, the average of FN was 3.69 ± 7.96% for the HVF and 0% for the VRP. The FP rate was 4.22 ± 5.91% and 0.83 ± 1.94% for the HVF and the VRP, respectively (Table 2). Specifically, 92.2% of FP with the HVF were below 15%, while 7.8% had a FP rate ≥ 15%. In contrast, all FP with the VRP (100%) were < 15%. The FN rate was lower than 33% in 99% of eyes with the HVF and in 100% with the VRP. The FL were ≤ 20% in 74.15% of eyes with the HVF and in 85.55% of eyes with the VRP (Table 2).

Finally, the time duration for both VF testing modalities is summarized in Table 2, with a significantly shorter test duration for the VRP device. The mean time duration of the test per eye on the HVF was 256.8 ± 7.1 s (4.28 ± 0.12 min), and 124.31 ± 0.35 s (2.07 ± 0.01 min) on the VRP (*P*<.0001).

## Discussion

This study compares the performance of the VisuALL VRP with the technician-based eye examinations in assessing visual acuity, color vision, and visual fields in patients from a neuro-ophthalmology clinic. The BCVA and CV showed significant positive correlations and good agreement with the gold-standard exams. The VF MD and PSD parameters from the VRP statistically correlated with those from the traditional exam on HVF.

Several VR devices have been developed in recent years to improve clinical and surgical training in ophthalmology, enhancing the diagnosis and assessment of patients, and serving as therapy for amblyopia or low vision defects [2, 17]. To the best of our knowledge, this is the first study that analyzes the VA and CV results with a VR platform in patients with different neuro-ophthalmologic disorders. Up to date, hardly any VR devices have assessed VA [17, 18]. However, using mobile or computer applications for the clinical evaluation or self-assessment of VA [19–23] has grown significantly. Similarly to our results, digital or tablet-based systems such as DigiVis or iSight Professional and Peek Acuity have shown good agreement with the Snellen chart in healthy populations [23].

The clinically acceptable variation for VA was described as below ± 0.2 lines, with a bias close to zero, intervals not crossing the zero line, and a correlation coefficient greater than 0.7 [24]. Considering this, the BCVA results for the VisuALL VRP device used in this study showed a bias close to zero, and both the limits of agreement (LOA) and the correlation coefficient almost reached the previously described values. These minor discrepancies may arise because these margins were described for normal subjects [24] and due to the heterogeneity of our sample. Moreover, although the assessment of VA using single letters flanked by bars may differ from the typically used crowded lines [25, 26], our results were still comparable to those obtained with the Snellen chart, indicating that the distance between the letter and the flanks from the VRP was acceptable [26].

Some smartphone applications have also been developed to assess CV [27–30], but limited studies have explored the use of VR devices for this purpose [31]. Our results showed that the VisuALL VRP evaluates CV more interactively than the traditional Ishihara booklet while providing comparable results. Although the differences were minor compared to the standard test, they may be attributed to the size, contrast, brightness, and color saturation of the images presented, as proposed by some authors [27, 28].

Unlike VA and CV, the use of VRP for evaluating the VF has been widely implemented, particularly in the glaucoma management [32–35]. Some studies evaluating VF defects in patients with glaucoma using VR devices have demonstrated an excellent correlation between those and HVF [33, 36, 37]. In the field of neuro-ophthalmology, our study comprehensively compares the VisuALL VRP with the standard HVF, evaluating MD, PSD, reliability indexes, and time duration. Previous studies with this type of patients conducted a more limited analysis using different VR devices [38, 39]. They observed a positive correlation in the patterns of VF defects between the VRP and automated perimetry, but a comparison of the MD or PSD across methods was not conducted [38, 39].

Based on our overall results, the VF evaluation using the VRP shows good agreement with the standard HVF. The data points outside the limits of agreement in the MD may be associated with the presence of the different conditions included within the neuro-ophthalmology clinic.

Subjects generally perform better on the test with the VRP compared to the standard HVF according to the reliability indexes. However, it is important to note that the methodology for assessing the reliability indexes varies between devices. Specifically, the differences in the FP and FN parameters may be attributed to the way these machines measure them and the fact that patients may perform better on the test because they feel more comfortable during the process [32]. Furthermore, one of the main advantages of this VRP is the simultaneous assessment of each eye during the VF testing. This method drastically saves time and improves patient performance while analyzing 30 degrees of the VF. The duration of HVF appears to be consistent across studies, regardless of population differences, with values ranging between 4 and 6 min per eye [40, 41] whereas the VF assessed using this VRP showed values of approximately 2 min per eye. Moreover, the ability to study both eyes simultaneously not only reduces time but also enables the objective detection of functional VF losses, as the patient is unaware of which eye is being stimulated.

Regarding the fixation losses (FL), results could not be compared statistically since the two devices calculate gaze fixation differently. The VisuALL VRP features a dynamic matrix algorithm that accounts for microsaccades. The VRP continuously tracks the center of the pupil, adjusting the fixation point and the stimulus matrix as the patient moves their eyes, reducing their FL. This innovative feature enhances the acquisition of VF, even in patients undergoing the process for the first time, and improves reliability indexes. Despite the differences in measuring the reliability indexes, patients produced reliable results on both devices (74.15% of eyes or more had less than 20% FL).

Additionally, the comfort of VRP has enabled the expansion of the patient population capable of undergoing VF assessment, obtaining reliable results even in pediatric patients [4, 42]. While some studies reported acceptable and reliable results in children between 8 and 10 years old using HVF, their attention span limits their performance [4]. The game-like format provided by the VisuALL VRP for assessing the visual field offers an engaging and motivating experience for children as young as 6 years old [42]. In this regard, not only pediatrics but also hospitalized, wheelchair-bound, bedridden, and overweight patients can perform a VF test while wearing the VRP device. It is well known that increased patient comfort, along with reduced test duration and fatigue, improves VF performance and test reliability [43]. Moreover, patients can wear their prescription glasses if needed because the device does not require an eye patch or trial lenses, which increases comfort during the test. On the other hand, the guidance provided by the AI virtual assistant during the test allows the specialized technician to use time more efficiently, ensuring a smoother clinical workflow.

Nevertheless, some limitations exist with the use of VRP. Some patients lack sufficient technical skills, which leads to poor performance in some individuals and the exclusion of some eyes. These results could have been improved if patients had undergone prior training with the VR device. The use of the VRP device requires minimal ambient noise and distractions. Moreover, future studies should assess the role of binocular vision when using the device [44], especially during VF assessments. When both eyes are tested simultaneously, the presence of strabismus or phorias may lead to fusion loss. This can negatively affect test performance. However, the device can also be set to test each eye in these cases independently.

In conclusion, this study assesses the VisuALL VRP platform in a neuro-ophthalmology clinic, and demonstrates that its performance is reliable and comparable to technician-based examinations for visual acuity, color vision, and visual field assessment, while requiring significantly less time. The reduced time and the guidance provided throughout the test by the AI virtual assistant help ensure optimal patient performance. As a result, this compact, wearable, and easy-to-use device has the potential to improve clinical workflows, facilitate home monitoring, and enhance the overall patient experience.

## Data Availability

The data that support the findings of this study are available from the corresponding author upon reasonable request.

## Abbreviations

AI: Artificial intelligence
BCVA: Best corrected visual acuity
CV: Color vision
FDA: Food and Drug Administration
FL: Fixation losses
FN: False negatives
FP: False positives
HIPAA: Health Insurance Portability and Accountability Act
HMD: Head-mounted-device
HVF: Humphrey visual field
LCD: Liquid crystal display
MD: Mean deviation
PSD: Pattern standard deviation
VF: Visual field
VRP: Virtual reality platform
VR: Virtual reality
